# Malaria epidemic outbreaks in the Democratic Republic of Congo, part I: cross-sectional survey in Mweka District

**DOI:** 10.5281/zenodo.10870408

**Published:** 2015-09-10

**Authors:** Célestin N. Nsibu, Dieudonné N. Mumba, Gauthier K. Mesia, Thierry L. Bobanga, Célestin de P. Manianga, Clarisse M. Mbo, Samuel M. Mampunza, Gaston L. Tona

**Affiliations:** 1 Department of Pediatrics, University of Kinshasa, Democratic Republic of Congo; 2 Department of Tropical Medicine, Parasitology and Entomology, University of Kinshasa, Democratic Republic of Congo; 3 National Institute of Biological Research, Parasitology, Ministry of Health, Democratic Republic of Congo; 4 Department of Clinical Pharmacology and Therapeutics, University of Kinshasa, Democratic Republic of Congo; 5 Department of Social Sciences, Anthropology, University of Kinshasa, Democratic Republic of Congo; 6 National Malaria Control Programme, Ministry of Health, Democratic Republic of Congo

## Abstract

**Background:**

A series of outbreaks of fever has previously been reported in the DR Congo. The occurrence of similar outbreaks in Mweka district presented the opportunity to investigate these occurrences.

**Materials and Methods:**

Health facilities and communities were visited. Permission was obtained to access to health records and a questionnaire was competed in the community. Blood samples for malaria, salmonellosis, Chikungunya, dengue and filovirus testing were obtained both in health facilities and the communities. Capture of mosquitoes and larvae in breeding sites was done and used bednets were collected. Excel, SPSS and Stats Direct were used for analyses of epidemiological data and malaria case management, with the Chi-square test and Fisher’s Exact test used for assessing relationships resulting from contingency table analyses.

**Results:**

An increase in the number of malaria cases beyond the expected number for the study period was observed in the two health districts located in the savannah zone (p<0.05) and in one health centre among sixteen located in the forest zone (p<0.05). In the health facilities and households visited (653 people), 141 persons had fever of which 82.2% was attributed to *Plasmodium falciparum* malaria. An incidence of 5.87% was recorded in the first half of 2013. Hundred and sixty patients (6.9%) died among 2,304 admitted for severe malaria in the three referral hospitals, 118 of them were children of under five years old. PCR testing of the blood samples obtained during home visits revealed malaria parasites in 63 (73.3%) of the 86 analysed samples. The test was negative for other parasites and bacteria and one dengue virus case was detected. *Anopheles gambiae* from Mweka were found to be resistant to permethrin using the WHO susceptibility test, with a knock down rate of ≤ 50% and mortality of ≤ 30%.

**Conclusion:**

These investigations confirmed epidemic outbreaks in Mweka District caused by malaria with a high mortality rate in children below five years of age.

## 1 Introduction

It is often not easy to clearly define a malaria epidemic when incident occur outside of potentially epidemic areas where environmental conditions are marginal for mosquito vector and malaria parasite development such as highlands or semi-arid regions. In stable malaria transmission settings it can be defined as a sharp increase in malaria incidence that significantly exceeds the normal inter-seasonal variation [1,2]. Outbreaks of epidemic malaria can therefore be regarded as breaking a normal epidemiological equilibrium.

The weakness of formal disease surveillance systems in sub-Saharan Africa to provide accurate data contributes to the belated recognition of malaria epidemics. Most African countries learn more by way of the popular press and the concerns of the communities than through the routine surveillance system [3-6]. For several months in 2012, a series of outbreaks of fever associated with abdominal pain and anaemia was reported in some provinces of the DR Congo (Oriental, Equateur and Kasai Oriental). Rapid assessment indicated co-morbidity of malaria, salmonellosis and malnutrition with high frequency in children [7]. This co-infection of *Plasmodium falciparum* and *Salmonella* species in children promotes the sudden onset of severe anaemia. However, the occurrence of such outbreaks with high lethality in other parts of the country, especially in Mweka administrative district in Kasai Occidental province, required additional investigations, which were conducted by a multidisciplinary team from the University of Kinshasa and the Ministry of Health. The aims of these investigations were to analyse data collected both from health facilities and the communities, to identify potential climatic and seasonal variations and to describe the habits of indigenous populations on the use of control tools such as long-lasting impregnated bednets (LLINs). This study reports data collected through the monitoring surveillance system combined with cross-sectional surveys in health facilities and affected communities.

## 2 Materials and methods

A multidisciplinary team that included a clinician, an epidemiologist, a parasitologist, an entomologist, a pharmacologist, an anthropologist and a community health worker trainer conducted the study.

### 2.1 Study design

Three health districts were surveyed and data collected through routine surveillance was analysed. In each of these health districts, a general referral hospital, two health centres and at least a cluster of 20 households around these health facilities were selected and their householder interviewed. The selection of the health facilities took population density into account as well as access to health services provided. A map of household locations was drawn based on the distribution of the population. A sample of 100 houses with children below five years of age or pregnant women was surveyed, 20 in Bulape health district located in the forest zone, 40 in Kakenga and 40 others in Mweka located in the savannah zone. Households were defined as a group of persons occupying the same house under one family head; occasional visitors were excluded. Formal consent was obtained before inclusion.

In the health facilities, a clinical approach based on case observation, evaluation of medical records and the analysis of biological samples was used. Fever was defined as an abnormally high body temperature above 38°C. The number of malaria cases for the previous 5 years (2008-2012) and those for the first five months of 2013 in these health facilities and their health district was recorded. In addition, data concerning fever in the two weeks preceding the survey and death of children below five years of age in the preceding six months was collected from the 100 households selected in the communities around the health facilities.

### 2.2 Sample collection

Blood samples were collected from 237 people of which 141 had fever (90 in health facilities and 51 in selected households). Cerebrospinal fluid culture (CSF) was also obtained in two patients with neurological features. On different collected samples, the following analyses were performed: malaria rapid diagnostic test (RDT), thin and thick smear for malaria, real time-polymerase chain reaction (RT- PCR) based on RNA for Salmonella, flavivirus, yellow fever virus, and Chikungunya), card agglutination test for trypanosomiasis (CATT), enzyme-linked immunosorbent assay (ELISA) for dengue fever, as well as blood and CSF cultures. For subsequent studies, blood was spotted on filter paper and serum kept in liquid nitrogen under conventional biosecurity.

### 2.3 Entomological sampling

Entomological collections consisting of mosquito sampling in households early in the morning and larvae from breeding sites were undertaken. Forty LLINs collected from selected households were kept for bio-efficacy studies. For each net, subsamples (30x30 cm) were taken from the roof, lower side and upper side of the net. Standard WHO cone bioassays were performed with adults reared from *Anopheles gambiae* larvae collected from breeding sites. Four cones were placed on each subsample and five non-blood fed, 2-3 day old females were introduced and exposed for 3 minutes before being held for 1 hr and observed for knockdown and then 24 hrs and observed for mortality (with classification as for WHO susceptibility tests). Mean knock down and mortality were calculated for each treatment group. Subsamples of untreated nets were assessed concurrently as controls. In addition, an environmental assessment was conducted to look for possible contributing factors to the epidemic outbreaks in the surveyed settings.

### 2.4 Data analysis

Statistical software used for analyses of epidemiological data and malaria case management were Excel, SPSS and Stats Direct, with the Chi-square test and Fisher’s Exact test used for assessing relationships resulting from contingency table analyses. A p-value of less than 0.05 was considered significant.

### 2.5 Ethical considerations

The scientific committee of the University of Kinshasa that acts as the ethics review board reviewed the protocol for this study. Participants who consented to participate in this study were invited to sign a formal consent form; their names and other information collected were kept confidential. Feedback to the provincial authorities, the MoH and its partners was given during dissemination meetings.

## 3 Results

### 3.1 Data from health districts

**Figure 1.**
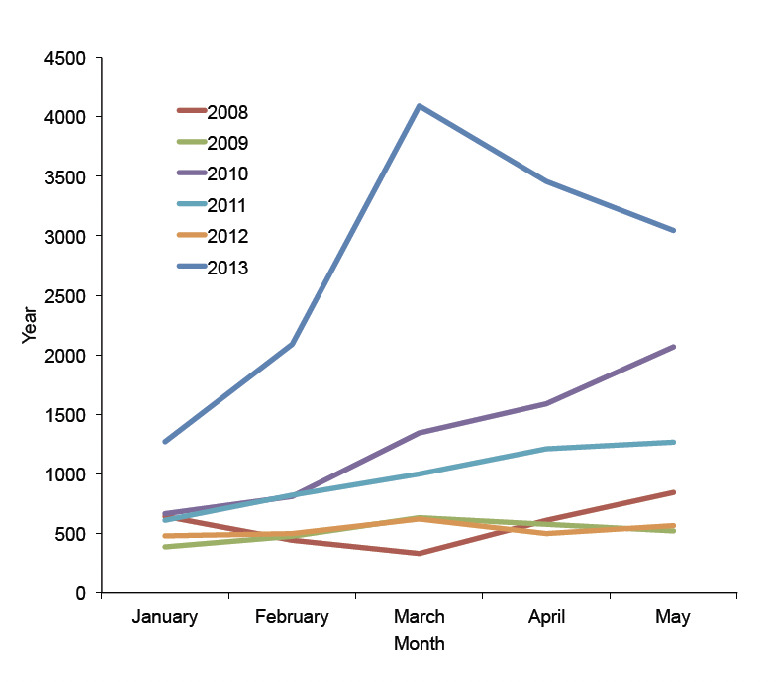
Malaria cases reported in the first five months of 2008-2013.

Data were grouped into two epidemiological scenarios according to their ecological similarity. Data from Kakenga and Mweka health districts located in the savannah were grouped and treated separately from those of Bulape, which is located in the forest. In the first group, we observed a sharp increase of malaria cases beyond the expected number for the period, as shown in Figure 1. Data of the first five months of 2013 were compared with the monthly mean number of cases and the mean plus two standard deviations (SD) calculated for the same periods from 2008 to 2012. An increase beyond the epidemic level was observed in Kakenga/Mweka health districts but not in Bulape (Table 1).

**Table 1.** Malaria cases detected by microscopy in the first five months of 2008-2012 versus 2013 in districts located in the savannah (Kakenga and Mweka) and forest (Bulape).

	January	February	March	April	May
**Kakenga/Mweka District**					
Mean number (2008-12)	558	610	785	898	1054
Mean number+2SD*	796	1369	2349	2805	3752
Reported cases (2013)	1272	2090	4098	3455	3051
**Bulape District**					
Mean number (2008-12)	792	610	785	914	994
Mean number+2SD	1355	1628	1658	1768	1818
Reported cases (2013)	732	913	805	659	915

* Mean number plus 2 standard deviations represents the epidemic level as in Cullen *et al.* [2].

There, while disaggregating data according to geographical characteristics of potentially epidemic settings, only one area of sixteen (Yoolo health centre) showed an increase of cases 5 times higher than what was expected between the 21^st^ to 23^rd^ epidemiological week of 2013.

In relation to the covered population, an incidence of 5.9% was recorded for the first half of 2013 in the health districts located in savannah.

### 3.2 Data from direct observation in health facilities

During the daily visits to the health facilities, 90 patients with fever including 43 males and 47 females were observed in the three general hospitals (38 cases) and the 9 health centres (52 cases). The median age was 8 years (7 months-55 years) with a predominance of children >5 years of age (54 versus 27, p <0.05). Of the 38 severe cases, malaria was observed in 30 (10 for 0-<5 yrs, 25 for 5-15 yrs, and 3 for >15 yrs). For the 52 non-complicated cases, malaria was observed in 44 (14 for 0-<5 yrs, 28 for 5-15 yrs, and 2 for >15 yrs). Thus, malaria was found in 74 patients (82.2%) with 3882±567 parasites/microliter; all were *Plasmodium falciparum*. No case of salmonellosis, Chikungunya, trypanosomiasis or dengue was found.

### 3.3 Community data

In the 100 households surveyed, 247 children <5 yrs old and 42 pregnant women were recorded. The prevalence of fever in children <5 yrs in the two weeks preceding the investigation was 39%. When considering all age groups in these surveyed households, 23% (150/653) experienced fever two weeks prior to the investigation. Among the families surveyed, 32 experienced at least one child death <5 yrs of age in the preceding 6 months, 42 deaths were recorded in total. These deaths mainly occurred during transfer to the general referral hospitals whereas specific hospital fatality due to malaria in children <5 yrs reached 5.2%, which corresponded to 118 deaths among 2304 patients admitted for severe malaria during the same period in the three referral hospitals of the 3 health districts.

Thin and thick smears for malaria were performed on 147 samples collected in the community (of which 51 from febrile patients); 43 samples (29.2%) were positive for *P. falciparum* (31 from febrile patients). According to the availability of reagents, RT-PCR was performed on only 86 blood samples to diagnose malaria, salmonellosis, Chikungunya or filovirus. Sixty-three samples (73.2%) were positive for malaria, none for the other pathogens.

Serological analysis for trypanosomiasis (CATT) was carried out on 84 samples of which 19 revealed an increase of antibodies titres (22.6%). These 19 samples were collected from children (mean age was 8 yrs, range 1-17 yrs) living in Kakenga health district. In addition, samples of febrile people (51) were analysed for dengue (ELISA); a 5 year-old child living in Yoolo health centre area in Bulape health district had a positive titre for dengue virus.

### 3.4 Entomological data

All the *Anopheles* specimens from Mweka district were identified as *An. gambiae s.s.* (M form, n=42). *Anopheles gambiae* from this setting was resistant to permethrin via WHO susceptibility tests, with a 1-hr knock down rate of 50% and 24-hr mortality of only 30%. Many breeding sites along roads under rehabilitation between Demba and Ilebo in Mweka district. In Kakenga district non-functional wells served as breeding sites. Near Bulape district breeding was observed where holes were drilled on the irregularities to facilitate climbing the outskirts of villages.

Ineffective use of impregnated nets distributed one year before was observed; most of these were not suspended due to the narrowness of the slots. Only 14/247 children <5 yrs slept under an LLIN the previous night and only 6/42 pregnant women did so. We also noted multiple holes on most of these nets.

## 4 Discussion

As reported by Guthmann *et al.* in 2007 [4], malaria epidemics remain poorly documented in sub-Saharan Africa partly because these occur in remote and rural settings areas where data collection is inefficient. The health information system in most of these countries is based on hospital data that provide an incomplete picture of the disease. Malaria is more prevalent in communities than observed in health facilities. Since most patients in low-resource settings do not seek medical care, estimates or extrapolations limit our understanding of what really occurs in the community.

Analysis of data collected in Kakenga/Mweka districts indicates that malaria epidemics were present in the community of these health districts six months before this investigation. An early warning system was not operational in these districts and surveillance was ineffective, which resulted in substantial delays in detection of this epidemic situation. This illustrates the weakness of the DR Congo surveillance system, which is not able either to predict or even describe such outbreaks in its population. A weekly detailed examination of collected data should be undertaken in health centres rather than waiting for accurate data analysis at district level which is currently carried out only once a month. Although the monthly validation meeting is taking part in each health district, the process is not putting emphasis on the detection of any outbreaks. The health district personnel is not trained for this purpose; it is only at the provincial level where a malaria officer is able to detect any outbreaks. Unfortunately, the provincial database is often not exhaustive and information not available on time.

A rapid assessment of collected data during the five years preceding this investigation in the Kakenga health district for instance shows an increase of number of malaria cases in the first five months of 2010. But this rise was not as high as that observed in 2013 which resulted in hospitalisations and deaths. The situation was completely different from the one observed in Bulape health district located in the forest where an abnormal increase of malaria cases was reported in only one health area, the Yoolo health centre area. This epidemic malaria outbreak was detected while disintegrating data of this health district, area by area. This suggests that data aggregation at the health district level obstructs detection of phenomena occurring in different health areas. Therefore it is necessary to analyse data first for each area separately before being transmitted to the health district level for analysis.

The analysis of data recorded in the health facilities proved that more malaria cases were observed in adults and children over 5 years of age than in younger patients. This fact suggests that a new phenomenon is occurring in the community during an epidemic outbreak and all age groups are affected. *Plasmodium falciparum* was the main cause of this epidemic outbreak because all other ailments such as salmonellosis, trypanosomiasis, chikungunya and other pathogens were not found apart from a dengue case observed in a five year-old boy living in Yoolo health centre area located in forest.

The cause of this re-emergence of a *P. falciparum* epidemic in this health district is controversial. Many factors such as climate changes and road’s constructional works are likely involved but the poor organisation of malaria control activities can be considered a major factor. Malaria case management was not appropriate because national guidelines were not implemented and recommended treatment was not available. For example, sulphadoxinepyrimethamine remains the first line treatment for uncomplicated malaria in two of the three health districts. These outbreaks occurred successively one year after the mass distribution of impregnated nets carried out in 2009 and 2011, respectively, which suggests that these protective tools were efficient only during the first year of their utilisation by the communities. In addition, malnutrition which is prevalent among young children in these settings has probably worsened clinical manifestations [8]. All these factors may have exacerbated the epidemics and contributed to excessive death rates observed.

Usually in these situations, hospital data don’t reflect the reality of what is happening in the community. The retrospective mortality survey coupled to the health facilities investigation found an important number of deaths among children <5 yrs of age in the previous six months. Due to the limitation of this retrospective survey, results should be interpreted with care [4]. Nevertheless it may reveal a new epidemiological scenario that occurred in these settings.

## 5 Conclusions

Our results confirm an epidemic outbreak of malaria in Kakenga/Mweka districts in 2013 and highlight the need to strengthen the routine data collection system, to develop early warning procedures and to translate evidence into timely actions to control these epidemics.
